# Outcomes of COVID-19 in people with rheumatic and musculoskeletal disease in Ireland over the first 2 years of the pandemic

**DOI:** 10.1007/s11845-022-03265-7

**Published:** 2023-01-09

**Authors:** Richard Conway, Elena Nikiphorou, Christiana A. Demetriou, Candice Low, Kelly Leamy, John G. Ryan, Ronan Kavanagh, Alexander D. Fraser, John J. Carey, Paul O’Connell, Rachael M. Flood, Ronan H. Mullan, David J. Kane, Frances Stafford, Philip C. Robinson, Jean W. Liew, Rebecca Grainger, Geraldine M. McCarthy, On behalf of the COVID-19 Global Rheumatology Alliance

**Affiliations:** 1 Department of Rheumatology, St. James’s Hospital, James Street, Dublin 8, Ireland; 2 Trinity College Dublin, Dublin, Ireland; 3 Department of Rheumatology, King’s College Hospital, London, UK; 4 Centre for RMDs, King’s College London, London, UK; 5 Department of Primary Care and Population Health, University of Nicosia Medical School, Nicosia, Cyprus; 6 Department of Rheumatology, St. Vincent’s University Hospital, Elm Park, Dublin 4, Ireland; 7 Department of Rheumatology, Mater Misericordiae Hospital, Dublin 1, Ireland; 8 Department of Rheumatology, Cork University Hospital, Wilton, Cork Ireland; 9 Galway Clinic, Galway, Ireland; 10 Department of Rheumatology, University Hospitals Limerick, Limerick, Ireland; 11 Graduate Entry Medical School, University of Limerick, Limerick, Ireland; 12 Department of Rheumatology, Galway University Hospitals, Galway, Ireland; 13 National University of Ireland Galway, Galway, Ireland; 14 Department of Rheumatology, Beaumont Hospital, Dublin 9, Ireland; 15 Royal College of Surgeons in Ireland, Dublin, Ireland; 16 Department of Rheumatology, Tallaght University Hospital, Dublin, Ireland; 17 Blackrock Clinic, Dublin, Ireland; 18 Faculty of Medicine, University of Queensland, Brisbane, Qld Australia; 19 Section of Rheumatology, Boston University School of Medicine, Boston, MA USA; 20 Department of Medicine, University of Otago, Wellington, New Zealand

**Keywords:** Biologics, COVID-19, Hospitalisation, RMD

## Abstract

**Background:**

Poor COVID-19 outcomes occur with higher frequency in people with rheumatic and musculoskeletal diseases (RMD). Better understanding of the factors involved is crucial to informing patients and clinicians regarding risk mitigation.

**Aim:**

To describe COVID-19 outcomes for people with RMD in Ireland over the first 2 years of the pandemic.

**Methods:**

Data entered into the C19-GRA provider registry from Ireland between 24th March 2020 and 31^st^ March 2022 were analysed. Differences in the likelihood of hospitalisation and mortality according to demographic and clinical variables were investigated.

**Results:**

Of 237 cases included, 59.9% were female, 95 (41.3%) were hospitalised, and 22 (9.3%) died. Hospitalisation was more common with increasing age, gout, smoking, long-term glucocorticoid use, comorbidities, and specific comorbidities of cardiovascular and pulmonary disease, and cancer. Hospitalisation was less frequent in people with inflammatory arthritis and conventional synthetic or biologic disease-modifying antirheumatic drug use. Hospitalisation had a *U*-shaped relationship with disease activity, being more common in both high disease activity and remission. Mortality was more common with increasing age, gout, smoking, long-term glucocorticoid use, comorbidities, and specific comorbidities of cardiovascular disease, pulmonary disease, and obesity. Inflammatory arthritis was less frequent in those who died.

**Conclusion:**

Hospitalisation or death were more frequently experienced by RMD patients with increasing age, certain comorbidities including potentially modifiable ones, and certain medications and diagnoses amongst other factors. These are important ‘indicators’ that can help risk-stratify and inform the management of RMD patients.

## Introduction

Infection with severe acute respiratory syndrome coronavirus 2 (SARS-CoV-2) and consequent Coronavirus disease 2019 (COVID-19) is an ongoing global health challenge. People with rheumatic and musculoskeletal disease (RMD) who develop COVID-19 may have worse prognosis compared to the general population [1]. The improvements in outcomes of COVID-19 that have occurred in the general population over time may not be seen in the RMD population [2–4]. Vaccination against SARS-CoV-2 offers protection in people with RMD; however, immunomodulating medications may prevent optimal vaccine responses in a small number of patients [5].

As public health restrictions lessen and increased interpersonal interactions occur in work and social settings, it becomes of increased importance to report data that people with RMD can use to understand the potential health implications. Exploration of the outcomes of COVID-19 in people with RMD and the associations with disease and individual specific factors may facilitate more precise/accurate risk assessments.

In this study, we report COVID-19 outcomes in people with RMD in Ireland over the first 2 years of the pandemic.

## Methods

### COVID-19 Global Rheumatology Alliance

Data regarding individuals with RMD with COVID-19 are entered into one of two parallel international data portals hosted in the USA and UK. Details of the C19-GRA registries have been published previously [6, 7].

### Data collection

Data input into the C19-GRA provider registry took place between 24th March 2020 and 31^st^ March 2022 and included baseline RMD status including demographic and clinical variables such as age, sex, smoking status, RMD diagnosis, disease activity (as per the physician's global assessment (remission, low, moderate, or high/severe)), and comorbidities. All diagnoses were physician-reported. Medications were categorised as previously described [8]. Disease-modifying antirheumatic drugs (DMARDs) were grouped as conventional synthetic DMARDs (csDMARDs) or biologic DMARDs (bDMARDs) and targeted synthetic DMARDs (tsDMARDs) [6]. RMDs were categorised as (1) inflammatory arthritis (IA) (rheumatoid arthritis, psoriatic arthritis, spondyloarthritis, juvenile idiopathic arthritis); (2) gout; (3) vasculitis, connective tissue diseases, and all other diagnoses (‘other’). Data collected regarding COVID-19 infection included method of diagnosis, place of diagnosis, COVID-19 symptoms, and outcomes of COVID-19 disease including hospitalisation, ventilation, and death.

Demographic and clinical continuous variables were reported as median (Interquartile range (IQR)) and categorical variables as number and percentage (%). Hospitalisation and mortality probability were calculated for each category of the key demographic and clinical variables. Differences in the likelihood of hospitalisation and mortality according to demographic and clinical variables were investigated using Chi-squared test or Fisher’s exact test, as appropriate.

All statistical analyses were performed and graphs were prepared using STATA IC15 (StataCorp. 2017. Stata Statistical Software: Release 15. College Station, TX: StataCorp LLC.).

This study was approved by the Irish National Research Ethics Committee for COVID-19 (20-NREC-COV-010). The committee waived the need for written informed consent as the data were anonymised.

## Results

Over the 2-year period, 237 cases of COVID-19 were reported in people with RMD (Table 1). The majority (59.9%) were female. Inflammatory arthritis (IA) was the most common diagnostic grouping (154/237, 65%), with the remainder having gout (35/237, 14.8%) and CTD/vasculitis/others (57/237, 24.1%). Most patients (46.5%) were in remission, 36.5% had low disease activity, 14.8% moderate disease activity, and 2.2% high disease activity. Eighty cases (33.8%) had no comorbidities. The most frequent comorbidities were cardiovascular disease (40.5%), pulmonary disease (16.9%), and obesity (9.7%). Of the 237 cases, 95 (41.3%) were hospitalised and 22 (9.3%) died.

**Table 1:** Outcomes according to demographic and clinical factors in people with RMD diagnosed with COVID-19

	**All participants (*n* = 237)**	**Not hospitalised (*n* = 135)**	**Hospitalised (*n* = 95)**		**Alive (*n* = 215)**	**Deceased (*n* = 22)**	
		**58.7%**	**41.3%**	**p-value**	**90.7%**	**9.3%**	***p*-value**
**Sex, *N* (%)**							
Female	142 (59.9)	87 (63.0)	51 (37.0)	0.101	130 (91.6)	12 (8.4)	0.589
Male	95 (40.1)	48 (52.2)	44 (47.8)		85 (89.5)	10 (10.5)	
**Age (years), *N* (%)**							
18–29	9 (3.8)	7 (87.5)	1 (12.5)	<0.001	9 (100.0)	0 (0.0)	<0.001a
30–49	59 (25.2)	47 (82.5)	10 (17.5)		59 (100.0)	0 (0.0)	
50–65	80 (34.2)	56 (71.8	22 (28.2)		75 (93.8)	5 (6.2)	
>65	86 (36.8)	23 (27.1)	62 (72.9)		69 (80.2)	17 (19.8)	
**Most common RMD diagnoses**^**a**^**, *N* (%)**							
Inflammatory arthritis^b^	154 (65.0)	108 (93.0)	40 (27.0)	<0.001	144 (93.5)	10 (6.5)	0.044
Gout	35 (14.8)	2 (5.7)	33 (94.3)	<0.001	27 (77.1)	8 (22.9)	0.007a
Connective tissue disease and other^c^	57 (24.1)	28 (50.9)	27 (49.1)	0.179	52 (91.2)	5 (8.8)	0.879
**Disease activity, *N* (%)**							
1 (Remission)	107 (46.5)	44 (42.3)	60 (57.7)	<0.001a	92 (86.0)	15 (14.0)	0.191a
2 (Low)	84 (36.5)	62 (75.6)	20 (24.4)		80 (95.2)	4 (4.8)	
3 (Moderate)	34 (14.8)	25 (73.5)	9 (26.5)		31 (91.2)	3 (8.8)	
4 (Severe/high)	5 (2.2)	1 (20.0)	4 (80.0)		5 (100.0)	0 (0.0)	
**No comorbidities, *N* (%)**	80 (33.8)	67 (85.9)	11 (41.3)	<0.001	79 (98.8)	1 (1.2)	0.002
**Most common comorbidities, *N* (%)**							
Cancer	10 (4.2)	1 (10.0)	9 (90.0)	0.002a	8 (80.0)	2 (20.0)	0.235a
Cardiovascular disease^d^	96 (40.5)	28 (29.8)	66 (70.2)	<0.001	77 (80.2)	19 (19.8)	<0.001a
Pulmonary disease^d^	40 (16.9)	12 (30.0)	28 (70.0)	<0.001	29 (72.5)	11 (27.5)	<0.001a
Neurological/neuromuscular/psychiatric disease	10 (4.2)	3 (30.0)	7 (70.0)	0.061a	8 (80.0)	2 (20.0)	0.235a
Obesity	23 (9.7)	13 (56.5)	10 (43.5)	0.823a	17 (73.9)	6 (26.1)	0.011a
**Smoking status, *N* (%)**							
Never	134 (67.3)	84 (63.2)	49 (36.8)	0.001	124 (92.5)	10 (7.5)	0.042
Ever	65 (32.7)	25 (39.1)	39 (60.9)		54 (83.1)	11 (16.9)	
**Medication prior to COVID-19 diagnosis, *N* (%)**							
Steroids	39 (83.5)	16 (42.1)	22 (57.9)	0.023	32 (82.1)	7 (17.9)	0.041
Steroids 10 mg or more	16 (6.8)	5 (33.3)	10 (66.7)	0.039	13 (81.3)	3 (18.7)	0.177
csDMARD monotherapy^e^	113 (47.7)	49 (70.0)	21 (30.0)	0.021	68 (93.2)	5 (6.8)	0.389
b/tsDMARD (monotherapy or in combination with csDMARD)^f^	92 (38.8)	66 (72.5)	25 (27.5)	0.001	86 (93.5)	6 (6.5)	0.243
**No complications, *N* (%)**	187 (78.9)	132 (71.7)	52 (28.3)	<0.001	180 (96.3)	7 (3.7)	<0.001
**Most common complications, *N* (%)**							
ARDS	10 (4.2)	0 (0.0)	9 (100.0)	<0.001a	3 (30.0)	7 (70.0)	<0.001a
Sepsis	9 (3.8)	0 (0.0)	9 (100.0)	<0.001a	4 (44.4)	5 (55.6)	<0.001a
Concomitant Infection	14 (5.9)	0 (0.0)	14 (100.0)	<0.001a	9 (64.3)	5 (35.7)	0.005a
Thromboembolism	11 (4.6)	0 (0.0)	11 (100.0)	<0.001a	10 (90.9)	1 (9.1)	0.729a
AKI or renal failure	7 (3.0)	0 (0.0)	7 (100.0)	0.002a	2 (28.6)	5 (71.4)	<0.001a
**Deceased, *N* (%)**	22 (9.3)	0 (0.0)	22 (100.0)	<0.001			

*P*-value from Pearson’s Chi square test, unless *a* = Fisher’s exact test

^a^Patients could be diagnosed with more than one RMDs

^b^Inflammatory Arthritis diagnosis includes: Axial spondyloarthritis (including ankylosing spondylitis) | Psoriatic arthritis | Other spondyloarthritis (including reactive arthritis) | Juvenile idiopathic arthritis, oligo | Juvenile idiopathic arthritis, poly | Systemic juvenile idiopathic arthritis | Rheumatoid arthritis | Other inflammatory arthritis

^c^Connective tissue disease and other diagnoses include: ANCA-associated vasculitis (e.g., GPA, EGPA) | other vasculitis including Kawasaki disease | anti-phospholipid antibody syndrome | autoinflammatory syndrome (including TRAPS, CAPS, FMF) | Bechet’s | chronic recurrent multifocal osteomyelitis | giant cell arteritis | IgG4-related disease | inflammatory myopathy (e.g., dermatomyositis, polymyositis) | inclusion body myositis (IBM) | mixed connective tissue disease | ocular inflammation | polymyalgia rheumatica | sarcoidosis | Sjogren’s syndrome | systemic lupus erythematosus | systemic sclerosis | undifferentiated connective tissue disease | localised scleroderma (morphea) | other

^d^Cardiovascular diseases include cerebrovascular disease, CVD, hypertension, diabetes and renal disease; pulmonary diseases include asthma, COPD and interstitial lung disease

^e^csDMARD monotherapy includes: Antimalarials (including hydroxychloroquine, chloroquine, mepacrine/quinacrine) | apremilast | azathioprine / 6-MP | | cyclosporine | leflunomide | methotrexate | mycophenolate mofetil / mycophenolic acid | sulfasalazine | tacrolimus | thalidomide / lenalidomide

^f^b/tsDMARD therapy includes: abatacept | belimumab | CD-20 inhibitors (including rituximab, ofatumumab) | cyclophosphamide | IL-1 inhibitors (including anakinra, canakinumab, rilonacept) | IL-6 inhibitors (including tocilizumab, sarilumab) | IL-12 inhibitors (ustekinumab) | IL 23 inhibitors (guselkumab, risankizumab) | IL-17 inhibitors (including secukinumab, ixekizumab) | JAK inhibitors (including tofacitinib, baricitinib, upadacitinib) | TNF-inhibitors (including infliximab, etanercept, adalimumab, golimumab, certolizumab, and biosimilars) | rituximab within the last 12 months

### Hospitalisation outcome

**Fig. 1:**
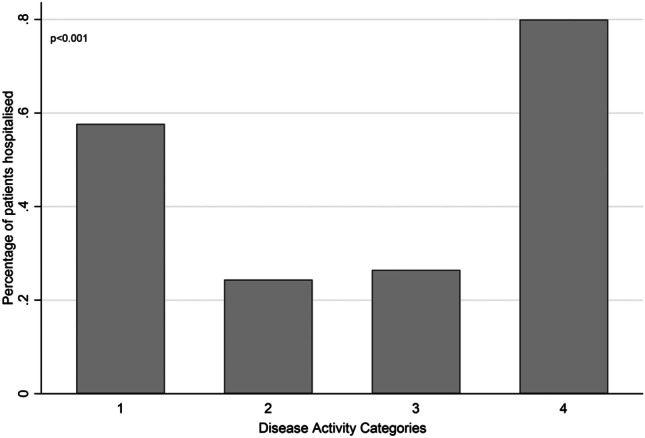
Frequency of hospitalisation by RMD disease activity

Demographic and clinical details according to hospitalisation are shown in Table 1. Hospitalisation was more frequently experienced with increasing age, a diagnosis of gout, smoking, long-term glucocorticoid use, comorbidities, and specific comorbidities of cardiovascular and pulmonary disease, and cancer. A diagnosis of inflammatory arthritis and conventional synthetic or biologic disease-modifying antirheumatic drug use were less common in those hospitalised. Hospitalisation appeared to have a *U*-shaped relationship with disease activity, being more common both in patients with high disease activity and those in remission (Fig. 1).

### Mortality outcome

Demographic and clinical details according to mortality are shown in Table 1. Mortality was more common with increasing age, a diagnosis of gout, smoking, long-term glucocorticoid use, comorbidities, and specific comorbidities of cardiovascular disease, pulmonary disease, and obesity. A diagnosis of inflammatory arthritis was less common in those who died.

## Discussion

This is the largest and most comprehensive study of outcomes of people with RMD and COVID-19 from Ireland. As reported in the general population, we found increasing age, comorbidities, specific comorbidities of cardiovascular and pulmonary disease, and obesity, and smoking to be positively associated with severe outcomes of hospitalisation or death. We have also identified potential RMD specific associations, with long-term glucocorticoid use and a diagnosis of gout more common, and a diagnosis of inflammatory arthritis, and conventional synthetic or biologic disease-modifying antirheumatic drug use less frequent in those with severe outcomes. RMD activity appeared to have a biphasic effect, with severe outcomes being more common in those with high disease activity and those with remission; this may be modulated by diagnostic group, particularly with gout patients (who otherwise had worse outcomes) being more likely to have disease activity labelled as “remission” between gout flares. However, the *U*-shaped relationship remained following exclusion of patients with gout.

The RMD-specific factors we have identified as being more frequent among RMD patients in Ireland with poor outcomes are broadly in keeping with previous studies in this area. Glucocorticoid use prior to the development of COVID-19, particularly in doses ≥ 10 mg/day have been associated with COVID-19-related hospitalisation and mortality [6, 9]. Increasing disease activity and its synergistic effect with increasing glucocorticoid doses has also been associated with more severe outcomes [9, 10]. Whether certain RMD groups or specific diagnoses within groupings are associated with worse outcomes is more difficult to ascertain with different studies reporting disparate findings [9, 11, 12]. Studies have demonstrated that specific medications such as rituximab, cyclophosphamide, and mycophenolate mofetil appear to be associated with more severe outcomes [9, 13, 14]. The potential for improved outcomes with individual medications or medication groups such as csDMARDs and bDMARDs is more controversial [8, 15].

Our study has several limitations. While this is the largest study of people with RMD and COVID-19 from Ireland, the statistical power of our study is limited by the relatively low number of cases reported, prohibiting the undertaking of multivariable analyses. The findings of our study must be interpreted with due cognisance of the limitations of the C19-GRA registry, including selection bias, unmeasured confounders, and possible artefact related to the identification of milder cases over time as testing capacity expanded [16]. The C19-GRA is a physician-entered registry and is limited by selection bias with a likely tendency to report more severe cases. It is also a case-based registry with no denominator population; therefore, inferences cannot be drawn about the incidence of COVID-19 in people with RMDs. Additionally, the C19-GRA is by design restricted to people with RMD and COVID-19; therefore, comparisons cannot be made to people with non-RMD and COVID-19 nor to RMD in the absence of COVID-19. We have reported data previously from the TRACR study which utilised a rigorous case ascertainment methodology; these results are broadly in keeping with the overall results reported here and should ameliorate some of the concerns regarding potential selection bias [4, 17]. The COVID-19 vaccination programme in Ireland occurred in parallel with the collection of data in this study and may have impacted findings; however, we have previously demonstrated no change in outcomes over time [4].

In conclusion, we have identified both shared features with the general population and RMD-specific factors which are over-represented among patients with severe COVID-19 outcomes. These factors may aid early identification of patients with RMDs with poor prognosis when prioritising booster vaccination and when prescribing COVID-19 antiviral therapy.

## Data Availability

The data underlying this article are available on reasonable request to the corresponding author.

## Data sharing statement

Request for access to data from the registry should be made to the Data Access and Sharing Committee of the COVID-19 Global Rheumatology Alliance.
